# Plasma amyloid beta 42 is a biomarker for patients with hereditary, but not sporadic, cerebral amyloid angiopathy

**DOI:** 10.1186/s13195-023-01245-2

**Published:** 2023-06-03

**Authors:** Anna M. de Kort, H. Bea Kuiperij, Lieke Jäkel, Iris Kersten, Ingeborg Rasing, Ellis S. van Etten, Sanneke van Rooden, Matthias J. P. van Osch, Marieke J. H. Wermer, Gisela M. Terwindt, Floris H. B. M. Schreuder, Catharina J. M. Klijn, Marcel M. Verbeek

**Affiliations:** 1 Department of Neurology, Radboud University Medical Center, Donders Institute for Brain, Cognition and Behaviour, Radboud Alzheimer Centre, P.O. Box 9101, Nijmegen, 6500 HB The Netherlands; 2 Department of Neurology, Leiden University Medical Center, Leiden, the Netherlands; 3 Department of Radiology, Leiden University Medical Center, Leiden, the Netherlands; 4 Department of Laboratory Medicine, Radboud University Medical Center, Nijmegen, The Netherlands

**Keywords:** Cerebral amyloid angiopathy, Hereditary cerebral hemorrhage with amyloidosis Dutch type, Sporadic cerebral amyloid angiopathy, Blood, Biomarkers, Diagnosis, Plasma Aβ38, Plasma Aβ40, Plasma Aβ42

## Abstract

**Background:**

The diagnosis of probable cerebral amyloid angiopathy (CAA) is currently mostly based on characteristics of brain MRI. Blood biomarkers would be a cost-effective, easily accessible diagnostic method that may complement diagnosis by MRI and aid in monitoring disease progression. We studied the diagnostic potential of plasma Aβ38, Aβ40, and Aβ42 in patients with hereditary Dutch-type CAA (D-CAA) and sporadic CAA (sCAA).

**Methods:**

All Aβ peptides were quantified in the plasma by immunoassays in a discovery cohort (11 patients with presymptomatic D-CAA and 24 patients with symptomatic D-CAA, and 16 and 24 matched controls, respectively) and an independent validation cohort (54 patients with D-CAA, 26 presymptomatic and 28 symptomatic, and 39 and 46 matched controls, respectively). In addition, peptides were quantified in the plasma in a group of 61 patients with sCAA and 42 matched controls. We compared Aβ peptide levels between patients and controls using linear regression adjusting for age and sex.

**Results:**

In the discovery cohort, we found significantly decreased levels of all Aβ peptides in patients with presymptomatic D-CAA (Aβ38: *p* < 0.001; Aβ40: *p* = 0.009; Aβ42: *p* < 0.001) and patients with symptomatic D-CAA (Aβ38: *p* < 0.001; Aβ40: *p* = 0.01; Aβ42: *p* < 0.001) compared with controls. In contrast, in the validation cohort, plasma Aβ38, Aβ40, and Aβ42 were similar in patients with presymptomatic D-CAA and controls (Aβ38: *p* = 0.18; Aβ40: *p* = 0.28; Aβ42: *p* = 0.63). In patients with symptomatic D-CAA and controls, plasma Aβ38 and Aβ40 were similar (Aβ38: *p* = 0.14; Aβ40: *p* = 0.38), whereas plasma Aβ42 was significantly decreased in patients with symptomatic D-CAA (*p* = 0.033). Plasma Aβ38, Aβ40, and Aβ42 levels were similar in patients with sCAA and controls (Aβ38: *p* = 0.092; Aβ40: *p* = 0.64. Aβ42: *p* = 0.68).

**Conclusions:**

Plasma Aβ42 levels, but not plasma Aβ38 and Aβ40, may be used as a biomarker for patients with symptomatic D-CAA. In contrast, plasma Aβ38, Aβ40, and Aβ42 levels do not appear to be applicable as a biomarker in patients with sCAA.

## Introduction

Cerebral amyloid angiopathy (CAA), i.e., cerebrovascular accumulation of amyloid β (Aβ), is a common pathology in the elderly. Moderate-to-severe CAA pathology is found in almost a quarter of the elderly population [1]. Sporadic CAA (sCAA) is a major cause of intracerebral hemorrhage (ICH) and cognitive decline [1]. The best known hereditary form of CAA, Dutch-type (D-CAA), also known as hereditary cerebral hemorrhage with amyloidosis Dutch type (HCHWA-D), is an autosomal dominant disorder caused by a point mutation at codon 693 of the amyloid precursor protein (APP) gene on chromosome 21, which leads to cerebrovascular Aβ accumulation [2, 3]. The clinical symptoms and neuroradiologic findings are similar to those in sCAA, although starting at a younger age. In D-CAA, symptomatic hemorrhage usually occurs between the age of 45 and 55, and cognitive impairment may already start at age 40 [3].

The diagnosis of sCAA during life is based on the criteria for detection of manifestations of the disease by neuroimaging, which have been updated in 2022 to include strictly lobar (micro)bleeds, cortical superficial siderosis, convexity subarachnoid hemorrhage, and specific white matter abnormalities (severe visible perivascular spaces in centrum semiovale or white matter hyperintensities in a multispot pattern) [4]. Whereas these Boston criteria 2.0 have a sensitivity of 80% and a specificity of 85%, they have their limitations. The MRI markers are irreversible and not exclusive to CAA. Moreover, the sensitivity in patients without an ICH is only 55%, whereas patients with CAA may also present with symptoms other than those of ICH, including cognitive complaints and transient focal neurological episodes. Fluid biomarkers may complement the diagnostic imaging criteria and could aid in clinical trial selection, monitoring of disease progression and success of disease-modifying therapies in both patients with sCAA and D-CAA.

Cerebrospinal fluid (CSF) biomarkers, specifically CSF Aβ38, CSF Aβ40, Aβ42, and Aβ43, have been shown to be able to discriminate CAA patients and controls [5–7]. Blood biomarkers could be a more patient-friendly, accessible, and cost-effective alternative to lumbar puncture and CSF analysis. We have previously shown that cerebral microbleeds are associated with increased levels of plasma Aβ38, Aβ40, and Aβ42 in the population [8]. In addition, we demonstrated increased plasma Aβ40 levels in patients with microbleeds in a cohort of small vessel disease (SVD) patients [9]. Interestingly, others found *decreased* levels of plasma Aβ40 and Aβ42 in a small cohort of presymptomatic D-CAA patients [10].

The aim of our study was to examine if plasma Aβ38, Aβ40, and Aβ42 can act as biomarkers for D-CAA and sCAA. For this, we studied separate discovery and validation cohorts of (pre)symptomatic D-CAA patients and controls, and a cohort of sCAA patients and controls.

## Methods

### Participants

#### D-CAA patients for discovery

We defined a D-CAA and a control group for discovery which included 35 patients with D-CAA and 40 controls from the Leiden University Medical Center (LUMC). Patients and controls were prospectively recruited either in the context of the “EDAN” study (Early Diagnosis of Amyloid Angiopathy Network; 11 presymptomatic D-CAA patients, 13 symptomatic D-CAA patients, and 29 controls, between 2012 and 2013) [11] or the CAVIA study (cerebral amyloid angiopathy: vascular imaging and fluid markers of Amyloid deposition; 11 symptomatic D-CAA patients and 11 controls, between 2016 and 2017; see Table 1). The presymptomatic patients were age- and sex-matched with 16 “young controls” (median: 37 years, interquartile range (IQR): 34–45 years), and the symptomatic patients with 24 “older controls” (median: 58 years, IQR: 53–63 years). The inclusion criteria for D-CAA patients were informed consent to participate in the study, the availability of a plasma sample, and DNA analysis confirmation of the c.2077G > C mutation in the APP gene. Patients were considered symptomatic if they previously had one or more ICH(s). Control participants were recruited from the general public or participants’ spouses, family, or friends and did not carry the APP mutation, nor had a contra-indication for MRI. All participants underwent a Mini-Mental-State-Examination (MMSE).

**Table 1:** Characteristics and results of plasma and CSF analysis of discovery D-CAA and control group

	**Presymptomatic D-CAA (*n* = 11)**	**Young controls (*n* = 16)**	**Symptomatic D-CAA (*n* = 24)**	**Older controls (*n* = 24)**	***P*-value/adjusted *p*-value**^**§**^**, Presymp vs YC**	B* (95% CI)	***P*-value/adjusted *p*-value**^**§**^**,** **Symp vs OC**	B** (95% CI)	***P*-value/adjusted *p*-value**^**§**^** Presymp vs Symp**	B*** (95% CI)
**Age (years)**	35 [24–47]	37 [34–45]	55 [51–60]	58 [53–63]	0.61^a^	–	0.079^a^	–	< 0.001^a^	–
**Sex, F/M (*n*)**	8/3	11/5	11/13	10/14	0.82^b^	–	0.78^b^	–	0.14^b^	–
**MMSE**	30 [30–30]	29 [29, 30]	29 [27–30]; *n* = 23	29 [29, 30]	0.58^a^	–	0.03^a^	–	0.005^a^	–
**Plasma**										
**Aβ38 (pg/ml)**	12.3 [10.3–13.0]	17.2 [13.9–18.9]	12.1 [10.1–13.8]	16.1 [14.1–19.9]	< 0.001^a^/ < 0.001^§^	− 5.22 (− 7.81 to − 2.63)	< 0.001^a^/ < 0.001^§^	− 4.81 (− 7.04 to − 2.58)	0.96^a^/0.17^§^	0.55 (− 2.36 to 3.46)
**Aβ40 (pg/ml)**	132 ± 37	164 ± 28	132 ± 30	156 ± 27	0.019^c^/0.009^§^	− 35.56 (− 61.15 to − 9.97)	0.005^c^/0.01^§^	− 22.96 (− 40.31 to − 5.61)	0.98^c^/0.70^§^	− 0.35 (− 24.18 to 23.48)
**Aβ42 (pg/ml)**	20 ± 7	28 ± 4	21 ± 4	27 ± 5	< 0.001^c^/ < 0.001^§^	− 8.96 (− 13.11 to − 4.82)	0.001^c^/ < 0.001^§^	− 5.38 (− 8.13 to − 2.64)	0.38^c^/0.07^§^	1.70 (− 2.17 to 5.56)
**CSF**										
**Aβ40 (ng/ml)**	3.17 [2.78–3.75]; *n* = 4	3.72 [3.33–4.68]; *n* = 5	1.36 [1.25–1.54]; *n* = 9	4.49 [1.41–5.23]^†^; *n* = 3	0.29^a^	–	0.036^a^	–	0.003^a^	–
**Aβ42 (pg/ml)**	512 [414–601]; *n* = 4	1008 [847–1120]; *n* = 5	298 [251–357]; *n* = 9	1022 [785–1342]^†^; *n* = 3	0.016^a^	–	0.009^a^–	–	0.006^a^	–

Values are medians and [IQR] except for plasma Aβ40, plasma Aβ42 (mean ± SD), and sex (*n*)

*Abbreviations*: *95% CI* 95% confidence interval, *Aβ* amyloid beta, *B* unstandardized beta coefficient, *CMB* cerebral microbleeds, *D-CAA* Dutch type hereditary cerebral amyloid angiopathy, *F* female, *IQR* interquartile range, *M* male, *MMSE* Mini-Mental-State-Examination, *OC* older controls, *Presymp* presymptomatic D-CAA, *SD* standard deviation, *Symp* symptomatic D-CAA, *YC* young controls

^a^Mann-Whitney test

^b^Chi-square test

^c^Student’s *t*-test

^*^Unstandardized *B* from linear regression with plasma Aβ as a dependent variable and age, sex, and group (patients with asymptomatic D-CAA versus young controls) as independent variables, with controls as the reference category

^**^Unstandardized *B* from linear regression with plasma Aβ as a dependent variable and age, sex, and group (patients with symptomatic D-CAA versus older controls) as independent variables, with controls as the reference category

^***  ^Unstandardized *B* from linear regression with plasma Aβ as an independent variable and group (patients with presymptomatic D-CAA versus patients with symptomatic D-CAA) as an independent variable, with presymptomatic D-CAA as the reference category

^§^*P*-value adjusted for age and sex with linear regression

^†^Median and range (as opposed to the interquartile range)

#### D-CAA patients for validation

We also defined a validation D-CAA and a control group which included 26 patients with presymptomatic D-CAA and 28 patients with symptomatic D-CAA from the LUMC. The presymptomatic patients were age- and sex-matched with 39 “young controls” (median: 40 years, IQR: 35–51 years), and the symptomatic patients with 46 “older controls” (median: 59 years, IQR: 52–70 years) from the Radboud University Medical Center (RUMC; see Table 2). The D-CAA samples were collected in the context of a D-CAA natural history study of the LUMC (the AURORA study; between 2018 and 2020 [12]). The patients with D-CAA had a proven mutation or a medical history of ≥ 1 lobar ICH(s) and ≥ 1 first-degree relative(s) with D-CAA. The anonymized controls underwent diagnostic workup in the RUMC in order to quantify either methylmalonic acid or homocysteine (between 2020 and 2021). We excluded patients who underwent the latter investigations in the context of a coagulation workup after a stroke or myocardial infarction.

**Table 2:** Characteristics and results of plasma and CSF analysis of D-CAA patients and controls for validation

	**Presymptomatic D-CAA (*n* = 26)**	**Young controls (*n* = 39)**	**Symptomatic D-CAA (*n* = 28)**	**Older controls (*n* = 46)**	***P*-value/adjusted *p*-value**^**§**^**, Presymp vs YC**	*B* (95% CI)*	***P*-value/adjusted *p*-value**^**§**^**, Symp vs OC**	*B* (95% CI)**	***P*-value/adjusted *p*-value**^**§**^**, Presymp vs Symp**	*B* (95% CI)***
**Age (years)**	38 [33–51]	40 [35–51]	58 [51–64]	59 [52–70]	0.81^a^	–	0.47^a^	–	<0.001^a^	–
**Sex, F/M (*n*)**	17/9	24/15	14/14	29/29	0.75^b^	–	1.00^b^	–	0.25^b^	–
**MOCA**	28 [26–29]	N.A	27 [24–28]	N.A.	–	–	–	–	0.01^﻿a^	–
**Plasma**										
**Aβ38 (pg/ml)**	12.3 [9.66–13.2]	12.6 [9.02–15.0]	13.9 ± 2.94	15.8 ± 2.94	0.24^a^/0.18^§^	− 1.63 (− 4.02–0.75)	0.06^c^/0.14^§^	− 1.69 (− 3.95–0.57)	0.002^a^/0.19^§^	2.67 (1.05–4.29)
**Aβ40 (pg/ml)**	118 [95–130]	119 [98.1–133]	135 ± 24.9	144 ± 42.5	0.55^a^/0.28^§^	− 9.28 (− 26.03–7.47)	0.23^c^/0.38^§^	− 7.36 (− 24.02 to 9.29)	< 0.001^a^/0.30^§^	24.82 (10.68–38.97)
**Aβ42 (pg/ml)**	23.4 [20.9–26.7]	26.1 [21.9–29.2]	26.0 [23.2–28.8]	28.6 [24.1–33.9]	0.31^a^/0.63^§^	− 0.87 (− 4.48–2.74)	0.04^a^/0.03^§^	− 3.43 (− 6.58 to − 0.29)	0.10^a^/0.87^§^	0.53 (− 2.84–3.91)
**CSF**										
**Aβ38 (ng/ml)**	1240 [1023–1183]; *n* = 11	N.A	785 [670–1165]; *n* = 11	N.A.	–	–	–	–	0.023^a^/ 0.63^§^	–
**Aβ40 (ng/ml)**	2.34 [1.88–3.34]; *n* = 10	N.A	1.68 [1.24–2.12]; *n* = 11	N.A	–	–	–	–	0.016^a^/ 0.041^§^	–
**Aβ42 (pg/ml)**	108 [86–148]; *n* = 10	N.A	72 [60–92]; *n* = 11	N.A	–	–	–	–	0.076^a^/ 0.21^§^	–

Values are medians and [IQR] except for sex (*n*), and plasma Aβ38 and plasma Aβ40 in the symptomatic patients and older controls (mean ± SD)

*Abbreviations*: *95% CI* 95% confidence interval, *Aβ* amyloid beta, *B* unstandardized beta coefficient, *D-CAA* Dutch-type hereditary cerebral amyloid angiopathy, *F* female, *IQR* interquartile range, *M* Male, *NA* not available, *MOCA* Montreal Cognitive Assessment, *OC* older controls, *Presymp* presymptomatic D-CAA, *SD* standard deviation, *Symp* symptomatic D-CAA, *YC* young controls

^a^Mann-Whitney test

^b^Chi-square test

^c^Student’s *t*-test

^*^Unstandardized *B* from linear regression with plasma Aβ as a dependent variable, and age, sex, and group (patients with presymptomatic D-CAA versus young controls) as independent variables, with controls as the reference category

^**^Unstandardized *B* from linear regression with plasma Aβ as an dependent variable, and age, sex, and group (patients with symptomatic D-CAA versus older controls) as independent variables, with controls as the reference category

^***^Unstandardized *B* from linear regression with plasma Aβ as an independent variable and group (patients with asymptomatic D-CAA versus patients with symptomatic D-CAA) as an independent variable, with presymptomatic D-CAA as the reference category

^§^*P*-value adjusted for age and sex

#### sCAA cohort

For the sCAA control group, we included 22 patients with sCAA from the RUMC and 39 patients with sCAA patients from the LUMC. The RUMC sCAA samples were included in the context of the “BIONIC” study (BIOmarkers for cogNitive Impairment due to Cerebral Amyloid Angiopathy, see www.radboudumc.nl/BCS; *n* = 19, between 2018 and 2021), or the CAVIA study (*n* = 3, in 2016) [7]. The LUMC sCAA samples were included in the context of the “FOCAS” study (Following Sporadic CAA Study). The inclusion criteria for the sCAA patients were informed consent to participate in the study, a diagnosis of probable CAA according to the modified Boston criteria [13], and the availability of a plasma sample. We included 42 controls from the RUMC, with the inclusion criteria similar to those of the controls for the D-CAA validation cohort. These controls partly overlap with the controls for the symptomatic D-CAA patients, but also include other older controls in order to age-match with the sCAA patients.

### Plasma analysis

Participants underwent a venipuncture after informed consent. In case of a history of ICH, venipuncture was done at least 3 months after the symptomatic hemorrhage. EDTA plasma was collected in polypropylene tubes, centrifuged, aliquoted, and stored in polypropylene tubes at − 80 °C for all patients, at all locations. For all plasma analyses, the technician who performed the analysis was blinded for the clinical diagnosis, and patient and control samples were randomly analyzed to avoid bias.

Aβ38, Aβ40, and Aβ42 levels were quantified in the plasma using ELISAs (Euroimmun, Lübeck, Germany). All measurements were performed in duplicate. The coefficient of variation (CV) was < 20% for all duplicate measures, except for 4 Aβ38 measurements with a CV of 21–31%.

For all ELISAs, five quality control samples were included on each plate to correct for any inconsistencies between plates. These controls consisted of pooled EDTA plasma samples that were stored in aliquots at − 80 °C. For each analysis, a fresh aliquot was used.

### CSF analysis

Part of the EDAN participants (12 patients with D-CAA, 8 controls), part of the AURORA participants (22 patients with D-CAA), part of the FOCAS patients (9 patients with sCAA), and all RUMC CAVIA (3 patients with sCAA) and BIONIC patients (22 patients with sCAA) underwent a lumbar puncture.

CSF Aβ40 and Aβ42 in the EDAN samples were measured as described previously [6]. In the samples of the BIONIC, AURORA, and FOCAS patients, CSF Aβ40 and Aβ42, were quantified using the Lumipulse chemiluminescent immunoassay (Fujirebio, Gent, Belgium). CSF Aβ38 was quantified in the BIONIC samples using ELISA (IBL, Fujioka-Shi, Japan) [7]. CSF analyses were used to study the correlations with plasma analyses.

### MRI acquisition

EDAN, FOCAS, and AURORA participants underwent a 3-T MRI scan using a standard 32-channel head coil (Philips Medical Systems, Best, The Netherlands). This protocol included T1, T2, fluid-attenuated inversion recovery images (FLAIR), and T2*-weighted images (EDAN) and susceptibility-weighted imaging (SWI) (FOCAS and AUROA). Further details are described in [11, 12].

Patients with sCAA from the BIONIC study underwent a 3-T MRI scan using a 32-channel head coil (Siemens Magnetom Prisma, Siemens Healthineers, Erlangen, Germany). Participants were examined using a comprehensive protocol, and for the current study, the 3D multi-echo gradient echo T2*-weighted sequence (voxel size 0.8 × 0.8 × 0.8 mm), the 3D T2-weighted sequence (voxel size 0.8 × 0.8 × 0.8 mm), and the 3D fluid-attenuated inversion recovery (FLAIR) sequence (voxel size 0.8 × 0.8 × 0.8 mm) were analyzed. Magnitude and phase data from the multi-echo gradient sequence was processed to a SWI using the “Contrast-weighted, Laplace-unwrapped, bipolar multi-Echo, ASPIRE-combined, homogeneous, improved Resolution SWI” (CLEAR-SWI) method [14].

The CAVIA sCAA patients underwent an MRI scan in a clinical context. This was either a 1.5- or 3.0-T. MRI and protocols included a T2, FLAIR, and a T2* or SWI sequence.

### MRI analysis

In the EDAN patients and controls, the number of microbleeds was analyzed as described in [11]. In the AURORA, FOCAS, BIONIC, and RUMC CAVIA patients, the following small vessel disease markers were analyzed: Fazekas score, cerebral microbleeds (CMBs), cortical superficial siderosis (cSS), and enlarged perivascular spaces (EPVS). Further details on the rating of these markers in the EDAN participants can be found in [11], in [12] for the FOCAS and AURORA participants, and in [7] for the BIONIC and RUMC CAVIA patients.

Furthermore, we defined the total burden score of SVD in CAA, an ordinal scale based on the number or extent/severity of the following 4 markers: lobar microbleeds, cortical superficial siderosis, perivascular spaces in the centrum semiovale, and white matter hyperintensities [15].

### Data analysis

The Shapiro-Wilk test was used to analyze the normality of the data. If parameters were normally distributed, they were depicted as mean ± standard deviation and group differences were analyzed with Student’s *t*-test or an ANOVA. Otherwise, they were stated as medians with interquartile ranges, and differences were analyzed with the Mann-Whitney *U* test or a Kruskal-Wallis test. Sex frequency was analyzed by the chi-square test. When comparing the group differences, we also adjusted for age and sex by performing multiple regression analysis with the patient group, age and sex as independent variables. The unstandardized *B* coefficients with the 95% confidence interval (95% CI) of the aforementioned regression analysis and the *p*-values of the unadjusted and adjusted analysis were shown in Tables 1, 2, and 3. The adjusted *p*-values were described in the text and shown in the figures, except for the *p*-values of the comparison between patients with presymptomatic D-CAA and symptomatic D-CAA. Disease progression here is highly associated with age in these patients, and therefore, we considered the unadjusted *p*-values most appropriate.

**Table 3:** Characteristics and results of plasma and CSF analysis of sCAA patients and controls

	**sCAA (*n* = 61)**	**Controls (*n* = 42)**	***P*-value/adjusted *p*-value**	*B* (95% CI)*
**Age (years)**	72 ± 6	72 ± 6	0.94^a^	–
**Sex, F/M (*n*)**	27/34	21/21	0.56^b^	–
**MOCA**	26 [23–27]; *n* = 26	N.A.	–	–
**Plasma**				
**Aβ38 (pg/ml)**	19.5 [15.8–22.8]	17.1 [13.2–20.6]	0.077^a^/0.092^§^	1.92 (-0.32–4.17)
**Aβ40 (pg/ml)**	169 [153–195]	155 [137–192]	0.14^a^/0.64^§^	3.97 (− 12.71–20.64)
**Aβ42 (pg/ml)**	31.9 ± 8.13	32.1 ± 6.03	0.74^a^/0.68^§^	0.58 (− 2.16–3.33)
**CSF**				
**Aβ38 (pg/ml)**	2174 ± 850; *n* = 16	N.A.	–	–
**Aβ40 (ng/ml)**	6.36 ± 2.92; *n* = 30	N.A	–	–
**Aβ42 (pg/ml)**	336 [165–400]; *n* = 30	N.A	–	–

Values are medians and [IQR] except for age; plasma Aβ42, CSF Aβ38, and Aβ40 (mean ± SD,) and sex (*n*)

*Abbreviations*: *95% CI* 95% confidence interval, *Aβ* amyloid beta, *B* unstandardized beta coefficient, *sCAA* sporadic cerebral amyloid angiopathy, *F* female, *IQR* interquartile range, *M* male, *MOCA* Montreal Cognitive Assessment, *N.A.* not available, *SD* standard deviation

^a^Mann-Whitney test

^b^Chi-square test

^§^*p*-value adjusted for age and sex

^*^Unstandardized *B* coefficient from linear regression with plasma Aβ as a dependent variable, and age, sex, and group (patients with sCAA versus controls) as independent variables, with controls as the reference category

Spearman rank correlation (*r*_sp_) was used to evaluate the correlation between non-normally distributed variables and to reduce the influence of outliers. To determine the diagnostic accuracy of the plasma Aβ, we determined the area under the curve (AUC) using a receiver operating characteristic curve (ROC) with a 95% confidence interval (CI). Using partial correlation, the correlation between the plasma Aβ peptides with MRI markers and the SVD burden score was adjusted for age. We only determined the AUC for the Aβ peptides with a significant difference between patients and controls.

We used the software programs IBM SPSS Statistics for Windows, version 25.0 (Armonk, NY: IBM Corp) and GraphPad Prism 5.03 (La Jolla, CA).

### Ethical statement

Venipunctures and lumbar punctures were performed after informed consent from the patients and controls. This study was approved by the Medical Ethics Committee Arnhem-Nijmegen and Medical Ethics Committee of Leiden and was conducted in accordance with the Declaration of Helsinki.

## Results

### Discovery cohort: patients with D-CAA compared to controls

**Fig. 1:**
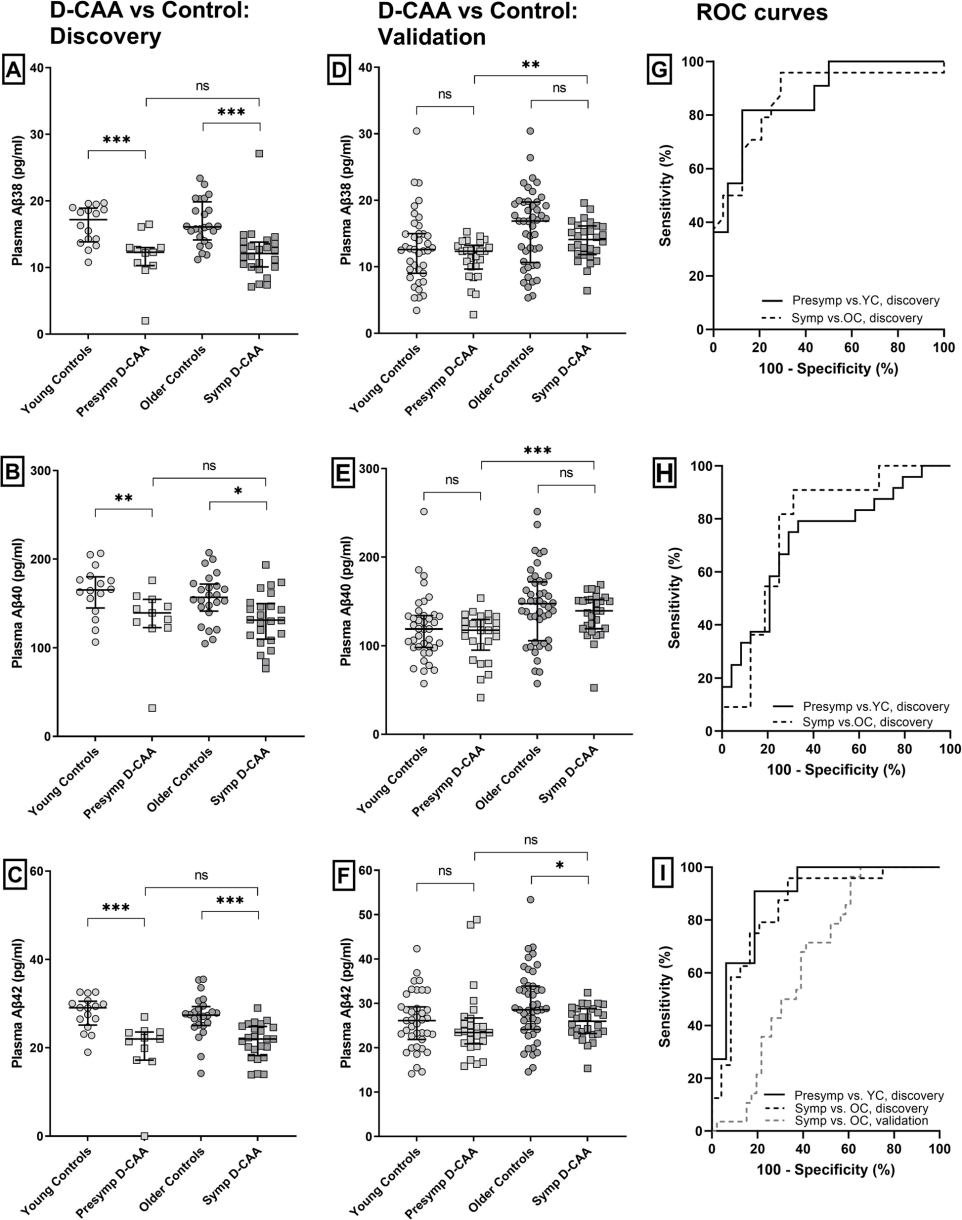
Plasma Aβ38, Aβ40, and Aβ42 levels in patients with D-CAA and controls for discovery, and patients with D-CAA and controls for validation. Scatterplots in all panels (depicting median and interquartile range). *p*-values are adjusted for age and sex. **A** Patients and controls for discovery. Plasma Aβ38 levels were significantly decreased in presymptomatic and symptomatic D-CAA patients versus their respective age-matched controls (both *p* < 0.001), but not in patients with symptomatic D-CAA versus patients with presymptomatic D-CAA (*p* = 0.96). **B** Patients and controls for discovery. Plasma Aβ40 levels were significantly decreased in patients with presymptomatic (*p* = 0.009) and symptomatic D-CAA (*p* = 0.01) versus their respective age-matched controls, but not in patients with symptomatic D-CAA versus patients with presymptomatic D-CAA (*p* = 0.98). **C** Patients and controls for discovery. Plasma Aβ42 levels were significantly decreased in patients with presymptomatic and symptomatic D-CAA versus their respective age-matched controls (both < 0.001), but levels in patients with presymptomatic D-CAA and symptomatic D-CAA were similar (*p* = 0.38). **D** Patients with D-CAA and controls for validation. Plasma Aβ38 levels were similar in presymptomatic (*p* = 0.18) and symptomatic D-CAA patients (*p* = 0.14) versus their respective age-matched controls. Levels were decreased in patients with presymptomatic D-CAA versus patients with symptomatic D-CAA (*p* = 0.002). **E** Patients with D-CAA and controls for validation. Plasma Aβ40 levels were similar in patients with presymptomatic (*p* = 0.28) and patients with symptomatic D-CAA (*p* = 0.38) versus their respective age-matched controls. Levels were decreased in patients with presymptomatic D-CAA compared to symptomatic D-CAA (*p* < 0.001). **F** Patients with D-CAA and controls for validation. Plasma Aβ42 levels were similar in patients with presymptomatic D-CAA and controls (*p* = 0.63) but decreased in symptomatic D-CAA patients (*p* = 0.033) versus controls. Levels in patients with presymptomatic D-CAA were similar to symptomatic D-CAA (*p* = 0.10). **G** ROC analysis of plasma Aβ38 yielded an AUC of 0.87 (95% CI 0.73–1.00; *p* = 0.001) to discriminate patients with presymptomatic D-CAA from controls and an AUC of 0.86 (95% CI 0.76–0.97; *p* < 0.001) to discriminate patients with symptomatic D-CAA from controls, in the discovery cohort. **H** ROC analysis of plasma Aβ40 yielded an AUC of 0.77 (95% CI 0.59–0.96; *p* = 0.018) to discriminate patients with presymptomatic D-CAA from controls and an AUC of 0.73 (95% CI 0.59–0.87; *p* = 0.006) to discriminate patients with symptomatic D-CAA from controls, in the discovery cohort. **I** ROC analysis of plasma Aβ42 yielded an AUC of 0.89 (95% CI 0.77–1.00; *p* = 0.001) to discriminate patients with presymptomatic D-CAA from controls and an AUC of 0.85 (95% CI 0.74–0.96; *p* < 0.0001) to discriminate patients with symptomatic D-CAA from controls, in the discovery cohort. In the validation cohort, ROC analysis of plasma Aβ42 yielded an AUC of 0.65 (95% CI 0.52–0.77; *p* = 0.036) to discriminate patients with symptomatic D-CAA from controls. *p < 0.05, **p < 0.01, ***p < 0.001. *Abbreviations*: AUC, area under the curve; D-CAA, Dutch-type cerebral amyloid angiopathy; Presymp D-CAA, presymptomatic D-CAA patients; Symp D-CAA, symptomatic D-CAA patients; OC, older controls; sCAA, sporadic CAA patients; ROC, receiver operator curve; YC, young controls

Plasma Aβ38, Aβ40, and Aβ42 levels were decreased in presymptomatic patients with D-CAA (Aβ38: *p* < 0.001; Aβ40: *p* = 0.009; Aβ42: *p* < 0.001) and symptomatic patients with D-CAA (Aβ38: *p* < 0.001; Aβ40: *p* = 0.01; Aβ42: *p* < 0.001) compared to their age-matched controls (Table 1; Fig. 1A–C). Similar plasma Aβ levels were found between presymptomatic and symptomatic D-CAA patients (Aβ38: *p* = 0.96; Aβ40: *p* = 0.98; Aβ42: *p* = 0.38).

**Fig. 2:**
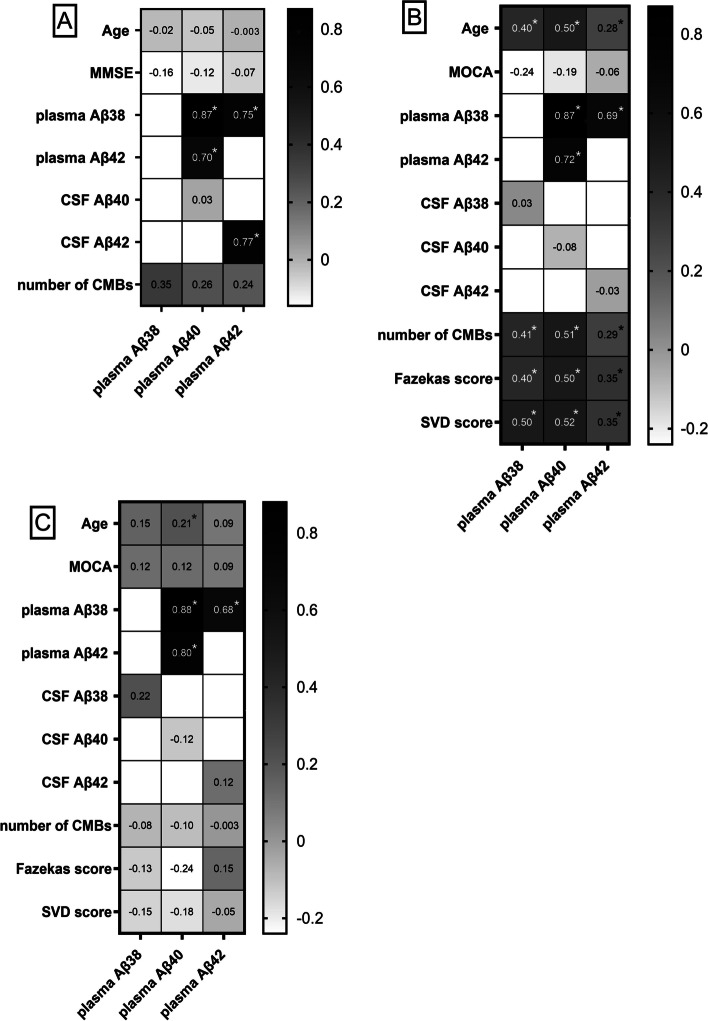
Correlation of plasma Aβ38, Aβ40, and Aβ42 with each other and other variables. Spearman correlation coefficients are stated. *A significant correlation (*p* < 0.05). **A** Correlation of plasma Aβ38, Aβ40, and Aβ42 with age, MMSE, other plasma Aβ peptides, CSF Aβ peptides, and number of strictly lobar microbleeds in the patients with D-CAA and controls of the discovery cohort. The correlation with MMSE and number of strictly lobar microbleeds was calculated in the patients with D-CAA only. MMSE (*n* = 34), CSF Aβ peptides (*n* = 21), and number of strictly lobar microbleeds (*n* = 23) were only available for a subset of patients. **B** Correlation of plasma Aβ38, Aβ40, and Aβ42 with age, MOCA, other plasma Aβ peptides, CSF Aβ peptides, number of strictly lobar microbleeds, Fazekas score, and small vessel disease burden score in the patients with D-CAA and controls of the validation cohort. The correlation with MOCA, CSF Aβ peptides, number of strictly lobar microbleeds, Fazekas score, and small vessel disease burden score was calculated in the patients with D-CAA only. MOCA (*n* = 56), CSF Aβ38 and Aβ42 (*n* = 22), CSF Aβ40 (*n* = 21), and number of strictly lobar microbleeds (*n* = 50), Fazekas score (*n* = 50), and small vessel disease burden score (*n* = 50) were only available for a subset of patients. **C** Correlation of plasma Aβ38, Aβ40, and Aβ42 with age, MOCA, other plasma Aβ peptides, CSF Aβ peptides, number of strictly lobar microbleeds, Fazekas score, and small vessel disease burden score in the patients with sCAA and controls. MOCA (*n* = 56), CSF Aβ peptides (*n* = 16 for CSF Aβ38, *n* = 30 for CSF Aβ40 and CSF Aβ42), and number of strictly lobar microbleeds (*n* = 57), Fazekas score (*n* = 59), and small vessel disease burden score (*n* = 57) were only available for a subset of patients. *Abbreviations*: CSF, cerebrospinal fluid; Aβ, amyloid beta; MOCA, Montreal Cognitive Assessment; SVD score, small vessel disease burden score

Plasma levels of Aβ38, Aβ40, and Aβ42 did not correlate with age, in combined D-CAA patients and controls, nor with MMSE score in the patients with D-CAA (Fig. 2A). All plasma Aβ peptides correlated with each other in the combined patients and controls (*r*_SP_ = 0.70–0.87; all *p* < 0.001; Fig. 2A). There was also a correlation between all plasma Aβ peptides in the patients with D-CAA and controls separately (D-CAA: Aβ38–Aβ40: *r*_SP_ = 0.89, Aβ38-Aβ42: *r*_SP_ = 0.57; Aβ40–Aβ42: *r*_SP_ = 0.62; controls: Aβ38–Aβ40: *r*_SP_ = 0.86; Aβ38–Aβ42: *r*_SP_ = 0.62; Aβ40–Aβ42: *r*_SP_ = 0.70, all *p* < 0.001). Plasma Aβ40 did not correlate with CSF Aβ40, but there was a correlation between plasma Aβ42 and CSF Aβ42 (*r*_SP_ = 0.77; *p* < 0.001; *n* = 21) in D-CAA patients and controls combined, but this correlation was not present in the patients with D-CAA (*r*_SP_ = 0.14; *p* = 0.65; *n* = 13). None of the plasma Aβ peptides correlated with the number of lobar microbleeds in the patients with D-CAA (Fig. 2A).

The AUCs of plasma Aβ peptides varied between 0.77 and 0.89 to discriminate patients with presymptomatic D-CAA from controls and between 0.73 and 0.86 to discriminate patients with symptomatic D-CAA from controls (For more details; see Fig. 1G–I).

### Validation cohort: patients with D-CAA compared to controls

Plasma Aβ38 and Aβ40 were similar in patients with presymptomatic D-CAA and controls (Aβ38: *p* = 0.18; Aβ40: *p* = 0.28) and in patients with symptomatic D-CAA (Aβ38: *p* = 0.14; Aβ40: *p* = 0.38) and controls (Table 2; Fig. 1D, E). Plasma Aβ42 was similar in patients with presymptomatic D-CAA and controls (*p* = 0.63) but decreased in patients with symptomatic D-CAA (*p* = 0.033) compared to controls. Plasma Aβ38 and Aβ40 levels in patients with presymptomatic D-CAA were decreased compared to patients with symptomatic D-CAA (Aβ38: *p* = 0.002; Aβ40: *p* < 0.001), and plasma Aβ42 levels were similar between patients with presymptomatic and symptomatic D-CAA (Aβ42: *p* = 0.10).

There was a correlation between age and plasma levels of Aβ38, Aβ40, and Aβ42 (*r*_SP_ = 0.28–0.50; all *p* < 0.001), in the combined patients and controls (Fig. 2B). All plasma Aβ peptides correlated with each other in the combined patients and controls (*r*_SP_ = 0.69–0.87; all *p* < 0.001; Fig. 2B). There was also a correlation between all plasma Aβ peptides in the patients with D-CAA and controls separately (D-CAA: Aβ38–Aβ40: *r*_SP_ = 0.85; Aβ38–Aβ42: *r*_SP_ = 0.51; Aβ40–Aβ42: *r*_SP_ = 0.58; controls: Aβ38–Aβ40: *r*_SP_ = 0.90; Aβ38–Aβ42: *r*_SP_ = 0.76, Aβ40–Aβ42: *r*_SP_ = 0.81; all *p* < 0.001). Plasma Aβ38, Aβ40, and Aβ42 levels and MoCA did not correlate in the patients with D-CAA (Fig. 2B). There was also no correlation between the plasma Aβ peptides and their CSF counterparts (Fig. 2B) in the patients with D-CAA. Furthermore, there was a correlation between plasma Aβ levels and the categorized number of lobar cerebral microbleeds, Fazekas score, and SVD burden score in the patients with D-CAA (*r*_SP_ = 0.29–0.52; *p* < 0.001–0.045; *n* = 50; Fig. 2B).

For plasma Aβ42, the only peptide for which a difference between the groups was observed in the validation cohort, we found an AUC of 0.65 (95% CI 0.52–0.77; *p* = 0.036) to discriminate patients with symptomatic D-CAA from controls (Fig. 1I).

### sCAA patients compared to controls

**Fig. 3:**
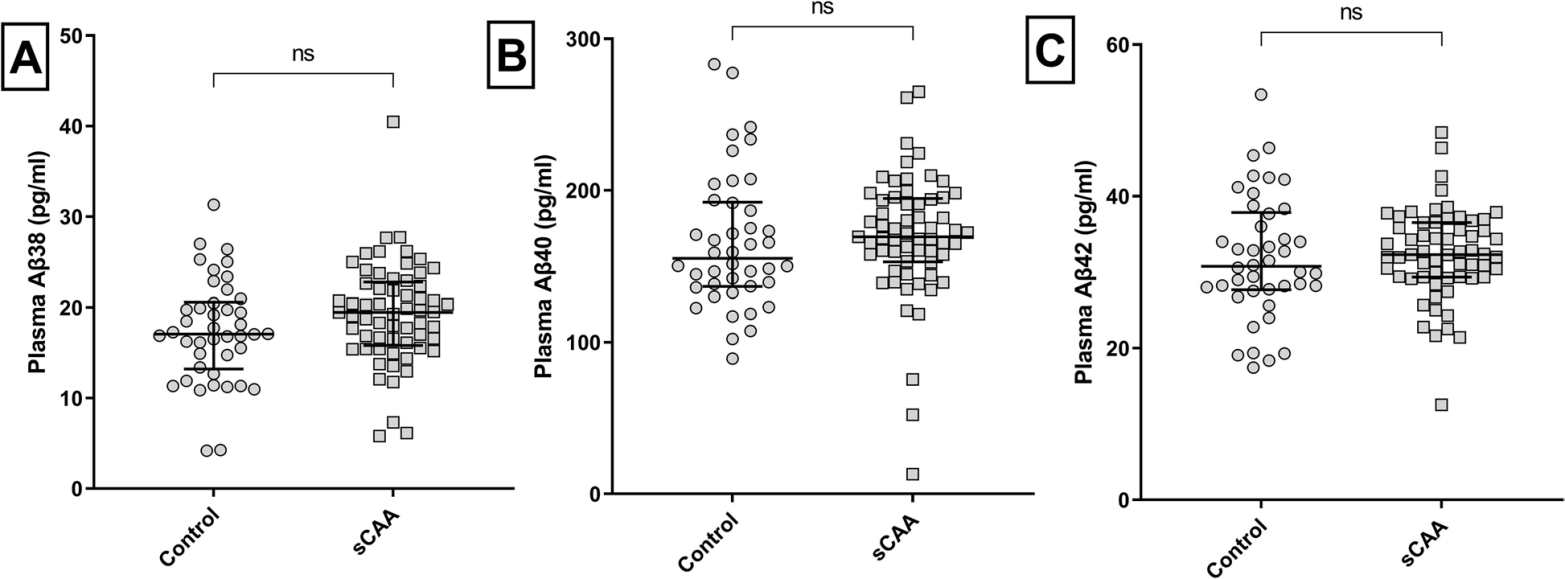
Plasma Aβ38, Aβ40, and Aβ42 levels in patients with sCAA and controls. Scatter plots in all panels (depicting median and interquartile range). *p*-values are adjusted for age and sex. **A** Plasma Aβ38 levels were similar in sCAA patients versus controls (*p* = 0.092). **B** Plasma Aβ40 levels were similar in sCAA patients and controls (*p* = 0.64). **C** Plasma Aβ42 levels were similar in sCAA patients and controls (*p* = 0.68). *Abbreviations*: *sCAA*, sporadic cerebral amyloid angiopathy *Ns*, non-significant

Plasma Aβ38, Aβ40, and Aβ42 levels were similar in patients with sCAA and controls (Aβ38: *p* = 0.092; Aβ40: *p* = 0.64, Aβ42: *p* = 0.68; Fig. 3).

Plasma Aβ38 and Aβ40 levels were significantly lower in patients with sCAA from the RUMC (Aβ38: *p* = 0.018; Aβ40: *p* = 0.029) compared to the patients with sCAA from the LUMC. Plasma Aβ42 was similar between the patients from both centers (*p* = 0.61). Plasma Aβ levels in patients with sCAA from the RUMC and controls from the RUMC were all similar.

There was a correlation between age and plasma Aβ40 (*r*_SP_ = 0.21; *p* = 0.03) but not with plasma Aβ38 or plasma Aβ42 in the entire cohort (Fig. 2C).

Plasma Aβ38, Aβ40, and Aβ42 levels and MoCA score did not correlate in the patients with sCAA (Fig. 2C). All plasma Aβ peptides correlated with each other in the combined patients and controls (*r*_SP_ = 0.67–0.88; all *p* < 0.001; Fig. 2C). There was also a correlation between all plasma Aβ peptides in the patients with sCAA and controls separately (sCAA: Aβ38–Aβ40: *r*_SP_ = 0.90; Aβ38–Aβ42: *r*_SP_ = 0.68; Aβ40–Aβ42: *r*_SP_ = 0.77; controls: Aβ38–Aβ40: *r*_SP_ = 0.83; Aβ38–Aβ42: *r*_SP_ = 0.64; Aβ40–Aβ42: *r*_SP_ = 0.82; all *p* < 0.001). There was no correlation between the plasma Aβ peptides and their CSF counterparts (Fig. 2C) in the patients with sCAA. Furthermore, there was no correlation between plasma Aβ peptides and categorized number of lobar cerebral microbleeds, Fazekas score, or SVD burden score in the patients with sCAA (Fig. 2C).

## Discussion

The main findings of our study were as follows: (1) In patients with symptomatic D-CAA, levels of plasma Aβ42 were significantly decreased, as consistently established in two independent cohorts. (2) Plasma levels of Aβ38, Aβ40, and Aβ42 were not consistently decreased in patients with presymptomatic D-CAA, and Aβ40 and Aβ42 were not consistently decreased in patients with symptomatic D-CAA, across two independent cohorts. (3) Levels of plasma Aβ38, Aβ40, and Aβ42 were similar in patients with sCAA and controls. (4) There was no correlation between plasma and CSF concentrations of either Aβ38, Aβ40, or Aβ42, in patients with D-CAA and sCAA.

Findings from previous publications regarding plasma Aβ levels in D-CAA have been inconsistent. One study found that plasma Aβ42 levels were decreased in patients with D-CAA (a pooled group of 15 patients with symptomatic D-CAA and 7 with presymptomatic D-CAA) compared to controls, whereas plasma Aβ40 levels were similar [16]. In contrast, another study reported a decrease of both Aβ40 and Aβ42 in patients with presymptomatic D-CAA (*n* = 9) compared to family members without the mutation (*n* = 8) [10]. Given these previous results, and since our study is the largest so far, we conclude that the decrease in plasma Aβ42 levels in patients with symptomatic D-CAA is the most robust finding across various studies. A possible application of plasma Aβ42 measurements in patients with symptomatic D-CAA may be (a more patient-friendly) monitoring of the efficacy of potential future disease-modifying drugs in clinical trials, although the AUC in the validation cohort was moderate (0.65). For CSF Aβ42, we found a higher diagnostic accuracy (AUC of 1.0 for patients with presymptomatic and symptomatic D-CAA versus controls [7]. Moreover, given the non-consistent findings in presymptomatic D-CAA, plasma Aβ42 cannot serve as an early biomarker. Furthermore, the fact that we could not internally replicate our results on Aβ38, Aβ40, and Aβ42 in two independent cohorts of presymptomatic D-CAA patients underlines the importance of replication of biomarker studies. This is further supported by a recent study that showed that the correlation between eight assays to measure plasma Aβ40 and Aβ42 in the same cohorts varied substantially: between *r* = 0.58–0.82 (for Aβ40) and between *r* = 0.21–0.81(for Aβ42).

We found similar levels of plasma Aβ38, Aβ40, and Aβ42 in patients with sCAA compared to controls. This is in accordance with an earlier study, where also no difference was found for plasma Aβ40 and Aβ42 levels between patients with sCAA (*n* = 25) and controls (*n* = 42) [17]. However, a second study found increased plasma Aβ40 and Aβ42 levels in patients with sCAA (*n* = 29; 8 patients with possible and 21 patients with probable sCAA) [18]. Given our own results and these reported inconsistencies, we conclude that plasma Aβ38, Aβ40, and Aβ42 are not reliable markers to detect sporadic CAA.

In D-CAA, the mutated form of Aβ, Aβ E22Q, aggregates faster into more stable and more proteolytic degradation–resistant fibrils than wild-type Aβ, and clearance across the blood-brain barrier is less efficient [19–21]. These processes lead to increased Aβ accumulation in the brain, which may then lead to lower levels of plasma Aβ42. This may, in part, explain why we detected decreased plasma Aβ42 levels in symptomatic D-CAA, but not in sCAA, compared to controls.

We did not find a correlation between plasma and CSF levels of the respective Aβ peptides in patients with sCAA and D-CAA. We only observed a correlation of plasma Aβ42 with CSF Aβ42 when data of controls and D-CAA of the discovery group was combined. In many previous publications, often in combined cohorts of controls, patients with mild cognitive impairment and AD [22–26], or in controls only [27], correlations between plasma and CSF Aβ40 and Aβ42 peptides were, at its best, weak (highest r for Aβ40: 0.20 [24] and Aβ42: 0.28 [26]). One study, however, found a negative correlation of *r* =  − 0.35 between plasma and CSF Aβ42 in patients with AD and a weakly positive correlation in controls (*r* = 0.19) [28]. Although we cannot exclude that we lack the power to detect a (small) association, we conclude that plasma Aβ38, Aβ40, and Aβ42 probably do not reflect the levels of their CSF counterparts, not only in our study on patients with sCAA and D-CAA, but also as a general observation. This may indicate that plasma Aβ levels are independent of cerebral Aβ metabolism and are predominantly influenced by peripheral processes, or may relate to the differences in the route of Aβ to blood versus to CSF. This may explain the limited biomarker value of plasma Aβ peptides to detect cerebral (vascular or parenchymal) Aβ accumulation. In accordance with this is the result of a meta-analysis of more than 50 studies, which did not show a convincing difference in plasma Aβ42 levels between patients with AD and controls [29], although some studies found that the plasma Aβ42:Aβ40 ratio can predict amyloid-PET status independent of clinical diagnosis [30, 31].

The limited biomarker value of plasma Aβ peptides to detect cerebral Aβ accumulation, and the lack of correlation between plasma and CSF Aβ levels, may also relate to the fact that levels of peripheral Aβ are affected by many processes. Peripheral Aβ may have several origins: cerebral Aβ can be transported to the circulation via the blood–brain-barrier (via receptors such as the LDL-related protein 1 (LRP1) receptor), arachnoid villi, or the glymphatic-lymphatic pathway [32]. In addition, Aβ can be produced in the periphery by platelets, skin fibroblasts, osteoblasts, and skeletal muscle cells [32]. Peripheral clearance of Aβ can take place via endocytosis or phagocytosis by white blood cells or hepatocytes, via excretion of bile or urine, and via Aβ-degrading enzymes. In addition, erythrocytes, albumin, antithrombin II, and lipoproteins such as ApoE also influence the concentration of Aβ in blood, by binding to Aβ [32]. Finally, there are many physiological factors that may influence plasma Aβ40 and Aβ42 levels: age (in our study we found inconsistent results: we only found a correlation with age and all plasma Aβ peptides in the validation cohort of patients with D-CAA and controls), renal and liver function (both affecting Aβ clearance), cerebrovascular diseases such as white matter hyperintensities and lacunes, coronary heart disease, body mass index, APOE ε4 status, and use of certain drugs such as acetylsalicylic acid, dipyridamole, antidiabetics, and anticoagulants [33–36]. Since increasing age is associated with more comorbidity and medication use, these factors may strongly affect the plasma Aβ levels, and interfere with group differences, especially in the patients with sCAA and their matched controls.

Pre-analytical conditions may also influence the results of plasma Aβ levels, such as the collected blood volume, the specific type of blood container, and the time between drawing and freezing of blood samples [37]. In addition, the used assay may influence the results: for example, conflicting results have been obtained with a Single Molecule Array (Simoa) platform and a xMAP platform: using the Simoa platform, significantly decreased plasma Aβ40 and Aβ42 levels were found between patients with presymptomatic D-CAA and controls, whereas in the same patients and controls, no differences were detected using the xMAP platform [10]. It has also been suggested that mass-spectrometry methods may be less prone to matrix effects in blood compared to immunoassays and thus may be more reliable to quantify plasma Aβ [26]. However, various mass-spectrometry methods also show variation in absolute measured values of plasma Aβ40 and Aβ42 [26].

Unknown technical factors may also play a role: one study found a difference between absolute levels of plasma Aβ in 70 samples that were measured twice, 4 years apart, using the same assay [35]. In addition, we found significantly higher levels of plasma Aβ38 and Aβ40 in the patients with sCAA from the LUMC compared to the patients with sCAA from the RUMC, implying a “center-effect,” despite using the same protocol for sample collection, processing and storage. We also found a center effect in CSF Aβ38, CSF Aβ40, and CSF Aβ42 measurements in patients with sCAA from the RUMC and LUMC [7]. Other groups have reported a center effect for CSF Aβ42 measurements as well [38, 39].

In the patients with D-CAA and controls for discovery, we found no correlation between plasma Aβ levels and the number of lobar microbleeds. However, in the validation cohort, we found a moderate correlation of all plasma Aβ peptides with the categorized number of microbleeds, Fazekas score, and the small vessel disease burden score (an ordinal score, based on the presence of four small vessel disease imaging markers associated with the severity of post-mortem CAA-associated vasculopathic changes [15]). This may indicate that plasma Aβ is associated with disease progression in D-CAA patients. The inconsistency between the two cohorts may be explained by a lack of power in the discovery cohort. We could, however, not find any relationship between any of the above markers with Aβ peptides in the patients with sCAA.

Strengths of this study include that we measured plasma Aβ38 levels (for the first time), Aβ40, and Aβ42 levels in two prospective, independent, extensively characterized, and largest to date, groups of patients with D-CAA and controls, and in a large group of patients with sCAA and controls.

## Limitations

Limitations include that we did not have cognitive and imaging data for part of the controls, we did not have information on APOE ε4 status, and other information that, based on earlier reports, may also influence Aβ levels such as medication use and kidney function. Lastly, in the validation cohort, we included patients from a different centers than the controls, which may also influence Aβ levels.

## Conclusions

Plasma Aβ42 was consistently decreased in symptomatic D-CAA patients across two cohorts, although we found inconsistent results with regards to AUC value in the two cohorts (0.89 and 0.65), which may limit biomarker potential. Plasma Aβ38 and Aβ40 lack the robustness to serve as a biomarker for patients with D-CAA. None of the plasma Aβ peptides can be used to detect sporadic sCAA.

## Data Availability

The datasets used and/or analyzed during the current study are available from the corresponding author upon reasonable request.
